# *Meso*-Dihydroguaiaretic Acid Ameliorates Acute Respiratory Distress Syndrome through Inhibiting Neutrophilic Inflammation and Scavenging Free Radical

**DOI:** 10.3390/antiox11010123

**Published:** 2022-01-06

**Authors:** Yen-Tung Lee, Yu-Li Chen, Yi-Hsuan Wu, Ih-Sheng Chen, Hsun-Shuo Chang, Yi-Hsuan Wang, Shih-Hsin Chang, Yi-Hsiu Wu, Ting-I Kao, Huang-Ping Yu, Tsong-Long Hwang

**Affiliations:** 1 Graduate Institute of Biomedical Sciences, Graduate Institute of Natural Products, College of Medicine, Chang Gung University, Taoyuan 333, Taiwan; tcm.6705@mmh.org.tw (Y.-T.L.); d0901501@mail.cgu.edu.tw (Y.-H.W.); fjord@cgmh.org.tw (T.-I.K.); 2 Department of Cosmetic Science, College of Human Ecology, Chang Gung University of Science and Technology, Taoyuan 333, Taiwan; 3 Department of Chinese Medicine, MacKay Memorial Hospital, Taipei 104, Taiwan; 4 Research Center for Chinese Herbal Medicine, Graduate Institute of Healthy Industry Technology, College of Human Ecology, Chang Gung University of Science and Technology, Taoyuan 333, Taiwan; ylchen03@mail.cgust.edu.tw (Y.-L.C.); yhwu03@mail.cgust.edu.tw (Y.-H.W.); d000016482@mail.cgu.edu.tw (S.-H.C.); ylwu02@mail.cgust.edu.tw (Y.-H.W.); 5 School of Pharmacy, College of Pharmacy, Kaohsiung Medical University, Kaohsiung 807, Taiwan; m635013@kmu.edu.tw (I.-S.C.); hschang@kmu.edu.tw (H.-S.C.); 6 Center for Molecular and Clinical Immunology, Chang Gung University, Taoyuan 333, Taiwan; 7 Division of Chinese Internal Medicine, Department of Traditional Chinese Medicine, Chang Gung Memorial Hospital, Taoyuan 333, Taiwan; 8 School of Traditional Chinese Medicine, College of Medicine, Chang Gung University, Taoyuan 333, Taiwan; 9 Department of Anesthesiology, Chang Gung Memorial Hospital, Taoyuan 333, Taiwan; 10 Department of Chemical Engineering, Ming Chi University of Technology, New Taipei City 243, Taiwan

**Keywords:** acute respiratory distress syndrome, *meso*-dihydroguaiaretic acid, neutrophil, reactive oxygen species, superoxide anion

## Abstract

The pathogenesis of acute respiratory distress syndrome (ARDS) is very complex. Patients with ARDS still suffer high mortality rates. Infiltration and activation of neutrophils in lungs are critical pathogenic factors in ARDS. In this study, we demonstrate that *meso*-dihydroguaiaretic acid (MDGA), a natural lignan, inhibits inflammatory responses in human neutrophils and ameliorates ARDS in mice. MDGA inhibited superoxide anion generation and elastase release in various G-protein coupled receptor agonists-induced human neutrophils. However, MDGA did not alter superoxide anion generation and elastase activity in cell-free systems. These results suggest that the anti-inflammatory effects of MDGA are mediated by regulating cellular signals in human neutrophils. In consistent with this, MDGA suppressed phosphorylation of extracellular signal-regulated kinase and *c*-Jun *N*-terminal kinase in activated human neutrophils. Moreover, MDGA inhibited CD11b expression and adhesion in activated human neutrophils. Interestingly, MDGA reduced reactive oxygen species (ROS) generation but not superoxide anion generation in protein kinase C (PKC) activator-induced human neutrophils, suggesting that MDGA may also have ROS scavenging ability. Indeed, MDGA showed strong free radical scavenging activity in cell-free assays. Significantly, MDGA suppressed PKC-induced neutrophil extracellular trap formation. Additionally, treatment of MDGA attenuated neutrophil infiltration and lung damage on lipopolysaccharide-induced ARDS in mice. In conclusion, our results demonstrate that MDGA has anti-neutrophilic inflammatory effects and free-radical scavenging activity. We also suggest that MDGA has potential to serve as a lead for developing new therapeutics to treat ARDS.

## 1. Introduction

Acute respiratory distress syndrome (ARDS) is a life-threating disease with a high mortality rate [1]. Severe acute respiratory syndrome coronavirus 2 (SARS-CoV-2) causes coronavirus disease 2019 (COVID-19) pandemic. ARDS is prevalent amongst patients with COVID-19 [2]. A previous paper reported that 41.8% of COVID-19 patients developed ARDS, and 52.4% of ARDS patients died [3]. Neutrophil accumulation in lung is a pathogenic marker of ARDS [4]. Neutrophil counts and activation are definitely correlated with the severity of ARDS in COVID-19 patients [5,6]. Neutrophils are recruited to the infected or inflamed lungs, and produce reactive oxygen species (ROS), release granules, and form neutrophil extracellular traps (NETs) to kill invading pathogens. However, unregulated cytotoxins produced by overactivated neutrophils cause tissue damage and lead to inflammatory lung diseases [7,8]. Excessive ROS produced by neutrophils leads to oxidative stress in the lungs [9,10]. Neutrophil elastase increases pulmonary permeability and pro-inflammatory cytokines enrolling more immune cells into the lung [11]. NETs contain decondensed chromatin fibers and granular proteases can destroy lung epithelium and endothelium [12]. Therefore, targeting neutrophil is a useful strategy to treat ARDS. However, limited pharmacologic therapy for ARDS is available [13].

Phytochemicals have gained increasing attention in the treatment of ARDS and COVID-19 [14]. Many herbal compounds have antiviral, anti-inflammatory, and antioxidant activities and can be used as adjunct therapeutics for virus-infected inflammatory disorders [15,16,17,18]. Lignans, a large group of phytochemicals, consist of propenylphenol units and demonstrate diverse bioactivities [19]. Antioxidant properties are commonly observed among lignans [20]. Other pharmacological activities such as anti-viral infection and anti-inflammatory disorders are also reported [21,22].

*meso*-Dihydroguaiaretic acid (MDGA), a dibenzylbutane-type lignan from *Machilus philippinensis* Merr., has several pharmacological effects, involving anti-inflammatory [23], anticancer [24], antibacterial [25,26], and neuroprotective effects [27,28]. Treatment with MDGA decreases mucus production in the respiratory tract to alleviate asthma [29]. MDGA reduced staurosporine-caused neurotoxic effect in primary mixed cortical cells via inhibiting ROS production [28]. MDGA is also a potential phytochemical for cardiovascular diseases as it inhibits vascular smooth muscle cell proliferation [30]. Furthermore, it was reported that ultraviolet-induced skin aging could be repaired by MDGA [31]. Nevertheless, the anti-inflammatory effect of MDGA against neutrophilic inflammation and ARDS is not reported before. In this study, we investigate the effects of MDGA on superoxide anion production, ROS generation, elastase release, cell adhesion, and NET formation in activated human neutrophils. Moreover, the protective effects of MDGA on lipopolysaccharide-induced ARDS in mice was evaluated.

## 2. Materials and Methods

### 2.1. Reagents

Ficoll–Hypaque was purchased from GE Healthcare (Chicago, IL, USA). Dextran was obtained from MP Biomedicals (Irvine, CA, USA). Extracellular signal-regulated kinase (ERK), phospho-ERK (Thr202/Tyr204), *c*-Jun *N*-terminal kinase (JNK), phospho-JNK (Thr183/Tyr185), Akt (pan), phospho-Akt (Ser473 & Thr308), p38, phospho-p38 (Thr180/Tyr182), Src, and phospho-Src family kinase (SFK) (Tyr416) antibodies were purchased from Cell Signaling Technology (Beverly, MA, USA). Leu-Glu-Ser-Ile-Phe-Arg-Ser-Leu-Leu-Phe-Arg-Val-Met (MMK-1) was obtained from Tocris Bioscience (Bristol, UK). 2,2′-Azobis(2-methylpropionamidine) dihydrochloride (AAPH), 2,2′-azino-bis(3-ethylbenzothiazoline-6-sulfonic acid) diammonium salt (ABTS), bovine serum albumin (BSA), cytochalasin B (CB), dihydrorhodamine 123 (DHR 123), dimethyl sulfoxide (DMSO), 1,1-diphenyl-2-picrylhydrazyl radical (DPPH), ferricytochrome *c*, fluorescein, *N*-Formyl-Met-Leu-Phe (fMLF), hydrogen peroxide solution, kolliphor EL, lipopolysaccharide (LPS), *o*-dianisidine dihydrochloride, phorbol 12-myristate 13-acetate (PMA), platelet-activating factor (PAF), polyethylene glycol, superoxide dismutase (SOD), vitamin E, Triton X-100, xanthine oxidase (XO), and xanthine sodium salt were purchased from Sigma-Aldrich (St. Louis, MO, USA). 2-(4-Iodophenyl)-3-(4-nitrophenyl)-5-(2,4-disulfophenyl)-2*H*-tetrazolium monosodium salt (WST-1) was obtained from Dojindo (Kumamoto, Japan). Interleukin-8 (IL-8) was obtained from ProSpec (East Brunswick, NJ, USA). Leukotriene B_4_ (LTB_4_) was purchased from Cayman Chemical (Ann Arbor, MI, USA). Elastase substrate and Trolox were obtained from Calbiochem (San Diego, CA, USA). Fluorescein isothiocyanate (FITC)-labeled anti-CD11b antibody, Hoechst 33342, horseradish peroxidase (HRP) anti-rabbit IgG, and SYTOX Green were purchased from Thermo Fisher Scientific (Waltham, MA, USA). Anti-neutrophil elastase antibody and HRP substrate were purchased from Millipore (Burlington, MA, USA). Nitrocellulose membranes were obtained from PerkinElmer Inc. (Boston, MA, USA). Anti-Ly6G antibody was obtained from eBioscience (San Diego, CA, USA). Antibodies for 4-hydroxynonenal (4-HNE), myeloperoxidase (MPO), and histone H3 (citH3) were purchased from Abcam (Cambridge, UK). bEnd.3 mouse brain cells were obtained from the Bioresource Collection and Research Centre (Hsinchu, Taiwan).

### 2.2. Extraction and Isolation

The dried root (7.6 kg) of *M. philippinensis* were extracted with methanol at room temperature. The MeOH extract was partitioned into ethyl acetate-soluble fraction, H_2_O-soluble fraction, and precipitate layer. The active ethyl acetate-soluble fraction (100 g) was chromatographed over silica gel using an *n*-hexane-ethyl acetate gradient to yield 18 fractions. Fraction 5 (0.74 g) was recrystallized and washed with *n*-hexane to obtain MDGA (183 mg). The structure of MDGA was determined by nuclear magnetic resonance (NMR, Mercuryplus-400/VNMRS-600 spectrometer, Varian, MA, USA), spectroscopic UV (U5100, Hitachi, Tokyo, Japan), infrared spectroscopy (FT/IR6000 FTIR spectrometer, Jasco, Kyoto, Japan), and mass spectroscopy (Bruker APEX II mass spectrometer, Burker, Karlsruhe, Germany).

MDGA presents as colorless needles in MeOH with an m.p. nearly to 80.5 °C. The chemical parameters of MDGA are as follows: [α]^24^_D_ ± 0 (*c* 0.20, CHCl_3_). UV λ_max_ (MeOH) (log ε): 228 (4.17), 281 (3.85) nm. UV λ_max_ (MeOH+KOH) (log ε): 214 (4.66), 246 (4.24), 296 (3.91). IR *v*_max_ (ATR): 3436 (OH), 1606, 1513 (aromatic ring) cm^−1^. EIMS *m/z* (rel.int. %): 330 [M]^+^ (31), 137 (100). ^1^H NMR (CDCl_3_, 200 MHz) *δ*: 0.84 (6H, d, *J* = 6.6 Hz, H-9, 9′), 1.75 (2H, m, H-8, 8′), 2.28 (2H, dd, *J* = 13.4, 9.1 Hz, H-7b, 7b′), 2.73 (2H, dd, *J* = 13.4, 5.0 Hz, H-7a, 7a′), 3.86 (6H, s, OCH_3_-3, 3′), 5.46 (2H, s, OH-4, 4′, D_2_O exchangeable), 6.62 (2H, d, *J* = 1.6 Hz, H-2, 2′), 6.67 (2H, dd, *J* = 7.8, 1.6 Hz, H-6, 6′), 6.82 (2H, d, *J* = 7.8 Hz, H-5, 5′).

### 2.3. Preparation of Human Neutrophils

This study was approved by the institutional review board of Chang Gung Memorial Hospital and written informed consent was acquired from all volunteers (aged 20–30 years). Following dextran sedimentation, neutrophils were isolated from venous blood by centrifugation using the Ficoll–Hypaque gradient technique with hypotonic lysis of red blood cells. The isolated neutrophils were evaluated using trypan blue assay, and >98% of viable cells were preserved in Hank’s balanced salt solution in ice-cold conditions until use [32].

### 2.4. Measurement of Superoxide Anion Production

Superoxide anion generation was assessed using ferricytochrome *c* reduction, as described in a previous study [33]. In brief, human neutrophils were incubated with ferricytochrome *c* (0.6 mg/mL) and DMSO (0.1%, as control) or MDGA (0.3–10 μM) at 37 °C for 5 min before stimulation. After priming with cytochalasin B (CB, 1 μg/mL) for 3 min, neutrophils were activated by adding fMLF (0.1 μM), MMK-1 (0.3 μM) for 10 min. Neutrophils were stimulated by adding PMA (10 nM) for 15 min without CB. Change in absorbance indicating ferricytochrome *c* reduction at 550 nm was continuously monitored in a spectrophotometer (U-3010, Hitachi, Tokyo, Japan).

### 2.5. Cytotoxicity Assay

The cytotoxicity of MDGA on human neutrophils was measured using a lactate dehydrogenase (LDH) assay kit (Promega, Madison, WI, USA). Human neutrophils were incubated with DMSO (0.1%; as control) or MDGA for 15 min. Total cellular LDH release was obtained by incubating Triton X-100 (0.1%) for 30 min. The absorbance at 492 nm were measured by Multiskan GO spectrophotometer (Thermo Fisher Scientific, Waltham, MA, USA). The DMSO and MDGA groups were compared with the Triton X-100 group, and the difference was represented as the total LDH release [34].

### 2.6. Intracellular Reactive Oxygen Species Determination

ROS reacted with DHR 123 to yield fluorescent rhodamine 123, which was detected to quantify ROS by flow cytometry (BD Accuri^TM^ C6, Biosciences, Cambridge, MA, USA). Neutrophils were loaded with DHR 123 (2 μM) for 12 min and then incubated with MDGA (1, 3, and 10 μM). After incubation for 5 min, the cells were activated by adding 0.1 μM fMLF with 1 μg/mL CB priming for another 5 min or by adding 10 nM PMA [34].

### 2.7. Total ROS Release Assays

ROS were measured by using luminol enhanced chemiluminescence. Human neutrophils (7 × 10^5^ cells/mL) were mixed with 37.5 μM luminol and 6 U/mL horseradish peroxidase (HRP) for 5 min, and then DMSO or MDGA (0.1–10 μM) were loaded 5 min before adding fMLF (0.1 μM) or PMA (10 nM). A 96-well chemiluminometer (Tecan, Infinite F200 Pro; Tecan Group, Männedorf, Switzerland) was applied to detect the chemiluminescence response [35].

### 2.8. 2,2′-Azobis(2-Methylpropionamidine) Dihydrochloride Scavenging Activity

MDGA or Trolox was preincubated with sodium phosphate buffer (75 mM; pH 7.4) and fluorescein (80 nM) at 37 °C. AAPH (25 mM) was loaded next, and changes in the fluorescence absorbance were measured every 3 min for 120 min by Tecan Infinite 200 PRO reader (Tecan, Männedorf, Switzerland). The excitation wavelength was 485 nm, and the emission wavelength was 535 nm [35].

### 2.9. Superoxide Anion Scavenging Activity

The scavenging extracellular superoxide effect of MDGA was examined in a cell-free xanthine/xanthine oxidase (XO) system. Neutrophils were incubated with Tris (50 mM; pH 7.4) assay buffer containing XO (0.02 U/mL) and WST-1 (0.3 mM) for 3 min in the presence or absence of MDGA (0.3–10 μM); superoxide dismutase (SOD) was used as the positive control. After adding 0.1 mM xanthine to the buffer, absorbance changes due to superoxide-induced WST-1 reduction were determined for 10 min at 450 nm at 30 °C by using a spectrophotometer (U-3010, Hitachi, Tokyo, Japan) [36].

### 2.10. Reactive Nitrogen Species Scavenging Activity

The scavenging effect of MDGA on reactive nitrogen species (RNS) radicals was demonstrated using DPPH and ABTS assays. In brief, MDGA was incubated with DPPH or ABTS, and changes in absorbance at 517 and 734 nm were measured by a spectrophotometer (U-3010, Hitachi, Tokyo, Japan). Vitamin E was used as a positive control for both DPPH and ABTS assays [35].

### 2.11. Evaluation of Elastase Release

Human neutrophils were first incubated with 100 μM elastase substrate (methoxysuccinyl-Ala-Ala-Pro-Val-*p*-nitroanilide) at 37 °C and then stimulated via the addition of fMLF, MMK-1, LTB_4_, IL-8, and PAF for 10 min in the presence of CB priming (0.5 or 2 μg/mL). Neutrophils were incubated with DMSO or MDGA for 5 min before stimulation [33]. Changes in absorbance at 405 nm were detected using a spectrophotometer (U-3010, Hitachi, Tokyo, Japan).

Additionally, human neutrophils were activated by fMLF (0.1 μM) with CB (1.5 μg/mL) for 15 min at 37 °C. After centrifuging at 1000× *g* for 5 min at 4 °C, the elastase supernatant was obtained. The supernatant was incubated with DMSO or MDGA at 37 °C for 5 min, and then elastase substrate (100 μM) was added. Changes in absorbance at 405 nm were continuously monitored for 10 min to evaluate elastase activity.

### 2.12. Assessment of CD11b Expression

Human neutrophils (5 × 10^6^ cells/mL) were preincubated with DMSO or MDGA at 37 °C and then stimulated via the addition of fMLF (0.1 μM) together with CB (1 μg/mL) for 5 min. After centrifugation at 4 °C, the cells were stained with FITC-labeled anti-CD11b antibody in 0.5% BSA for 90 min on ice. The fluorescence intensity was detected using flow cytometry (BD Accuri^TM^ C6, Biosciences, Cambridge, MA, USA) [33].

### 2.13. Neutrophil Adhesion Assay

bEnd.3 mouse brain cells were incubated with LPS (2 μg/mL) for 4 h. Human neutrophils were stained with Hoechst 33342 for 10 min before preincubation with DMSO or MDGA and then stimulated by adding fMLF (0.1 μM) together with CB (1 μg/mL) for 5 min. After co-culturing activated neutrophils and bEnd.3 cells for 30 min, non-adherent neutrophils were removed using HBSS; the remaining adherent neutrophils were counted manually in 3 randomly selected areas under a microscope (IX81, Olympus, Tokyo, Japan) with 10X objective [37].

### 2.14. NET Quantification

Neutrophils were incubated with DMSO or MDGA for 10 min and then activated by adding PMA (10 nM). After activation for 3 h, DNase (2 U/mL) was added at 37 °C for 10 min before stopping the reaction by adding EDTA at 4 °C. Afterward, supernatants were obtained, and SYTOX Green (5 μM) was added. Fluorescence changes were measured using the Tecan Infinite 200 PRO reader (Tecan, Männedorf, Switzerland) [35].

### 2.15. Immunofluorescence Staining of NETs

Human neutrophils were incubated on poly-L-lysine-coated glass coverslips for 30 min at 37 °C, and then incubated with DMSO or MDGA for 10 min before activated by adding PMA for 2 h. Neutrophils were fixed using paraformaldehyde for 15 min and lysed using Triton X-100 thereafter. The samples were first incubated with goat serum blocking buffer for 1 h and then with anti-elastase (5 μg/mL) and anti-myeloperoxidase (MPO, 5 μg/mL) antibodies before treatment with secondary antibodies. After washing with phosphate-buffered saline (PBS), the neutrophils were stained with Hoechst 33342, and images were assayed with a Zeiss LSM 510 META confocal microscope (Zeiss, Jena, German) [35].

### 2.16. Western blot Analysis

The neutrophils were incubated with DMSO or MDGA for 5 min before fMLF (0.1 μM) together with CB (1 μg/mL) addition. After activation for 30 s, a sample buffer was added to block the reaction at 100 °C for 15 min. Whole-cell lysates were obtained after centrifugation. Phosphorylation of mitogen-activated protein kinases (MAPKs), Akt, and Src family kinase (SFKs) was assessed by immunoblotting using corresponding secondary rabbit antibodies. Immunoreactive bands were visualized using HRP and evaluated using UVP Biospectrum (UVP, Upland, CA, USA) [37].

### 2.17. Lipopolysaccharide-Induced ARDS

BALB/c mice (male, 20–25 g, 7–9 weeks old) were purchased from BioLASCO (Taipei, Taiwan), and all animal experiments were approved by the Institutional Animal Care and Use Committee of Chang Gung University. The mice were randomly assigned into four groups: vehicle, MDGA only, LPS only, and MDGA with LPS. MDGA (30 mg/kg) dissolved in vehicle (10% DMSO, 20% kolliphor EL, and 70% polyethylene glycol) before use. The mice were intraperitoneally injected with MDGA or vehicle after anesthesia by isoflurane. After 1 h, ARDS was induced via intratracheal administration of LPS (2 mg/kg) or normal saline. The mice were anesthetized and sacrificed for obtaining the lung tissues after 6 h of LPS induction.

The left lung was immersed in 10% formalin for further histological and immunofluorescence observation. The lung tissues fixed using formalin were dehydrated and embedded in paraffin. The paraffin blocks were cut into 5 μm thick sections and embedded onto slides for staining with hematoxylin and eosin (HE), Ly6G antibody, or MPO antibody. The slides were then observed under a light microscope (IX81, Olympus, Tokyo, Japan) [38]. For immunofluorescence staining, tissue sections were incubated with antibodies citH3 (citrulline R2 + R8 + R17), Ly6G, and 4-HNE, respectively. Immunofluorescence images were acquired through BioTek LioHeart FX microscopy (Winooski, VT, USA).

The right lung was frozen at −80 °C to measure MPO activity. The lung tissues were ground in PBS and centrifuged to obtain a supernatant. The supernatant was diluted with PBS containing *o*-dianisidine dihydrochloride (0.167 mg/mL) and H_2_O_2_ (0.0005%). Light absorbance was measured spectrophotometrically at 405 nm by Multiskan GO spectrophotometer (Thermo Fisher Scientific, Waltham, MA, USA) and normalized to the corresponding protein concentration [38].

### 2.18. Statistical Analysis

Data are presented as box-and-whisker plots (median, min-max) or line plots (mean, standard error of the mean (SEM)). N values are independent experiments. Statistical analysis was performed using Student’s *t*-test (Prism, GraphPad Software 9.0.2, San Diego, CA, USA). *p* < 0.05 was considered statistically significant.

## 3. Results

### 3.1. MDGA Decreases Superoxide Anion Generation in fMLF- and MMK-1-, but Not PMA-Activated Human Neutrophils

**Figure 1:**
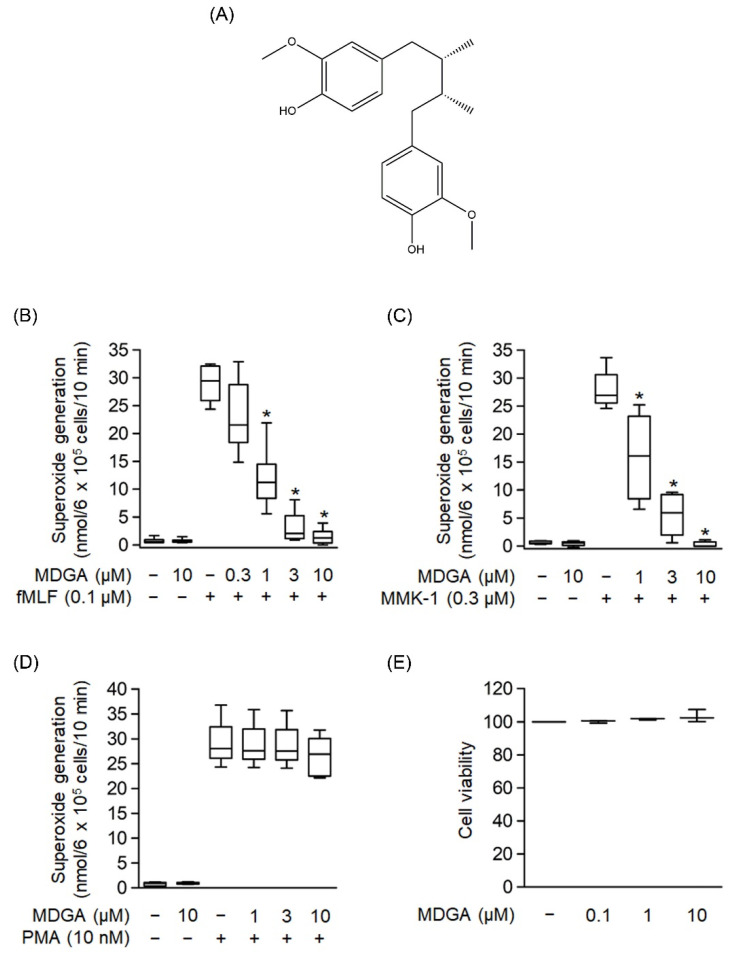
Effects of *meso*-dihydroguaiaretic acid (MDGA) on superoxide anion generation in human neutrophils. (**A**) Chemical structure of MDGA. (**B**–**D**) Superoxide anion generation was monitored using ferricytochrome *c* reduction. Visualization was carried out using a spectrophotometer at 550 nm after human neutrophils were incubated with dimethyl sulfoxide (DMSO, 0.1%, as control) or MDGA (0.3–10 μM) for 5 min, and thereafter stimulated by (**B**) *N*-formyl-Met-Leu-Phe (fMLF, 0.1 μM) + cytochalasin B (CB, 1 μg/mL) for 10 min, (**C**) Leu-Glu-Ser-Ile-Phe-Arg-Ser-Leu-Leu-Phe-Arg-Val-Met (MMK-1, 0.3 μM) + CB (1 μg/mL) for 10 min, or (**D**) phorbol 12-myristate 13-acetate (PMA, 10 nM) for 15 min, (*n* = 5 or 6). (**E**) Human neutrophils were incubated with DMSO (0.1%, as control) or MDGA (0.1, 1, and 10 μM) for 15 min. MDGA had no cytotoxicity in human neutrophils as revealed by LDH assay, (*n* = 3). The values are shown as the mean ± S.E.M. * *p* < 0.05 vs. stimulated control.

The chemical structure of MDGA is shown in Figure 1A. The generation of superoxide anion, a precursor of ROS, was analyzed to evaluate whether MDGA exerted an anti-inflammatory response. As shown in Figure 1B–D, human neutrophils were significantly activated by fMLF (0.1 μM), MMK-1 (0.3 μM), or PMA (10 nM). MDGA treatment showed concentration-dependent inhibitory effects on fMLF- and MMK-1-induced superoxide anion generation with IC_50_ values of 0.78 ± 0.17 μM and 1.17 ± 0.64 μM, respectively (Figure 1B,C). However, MDGA did not inhibit PMA-induced superoxide anion generation (Figure 1D), ruling out the effect of MDGA on the protein kinase C (PKC)-dependent pathway. In addition, MDGA treatment did not induce LDH release (Figure 1E) and failed to alter superoxide anion level in basal human neutrophils, suggesting that MDGA did not alter the cell viability and basal activity of human neutrophils.

### 3.2. MDGA Decreases ROS Production in fMLF- and PMA-Activated Neutrophils

**Figure 2:**
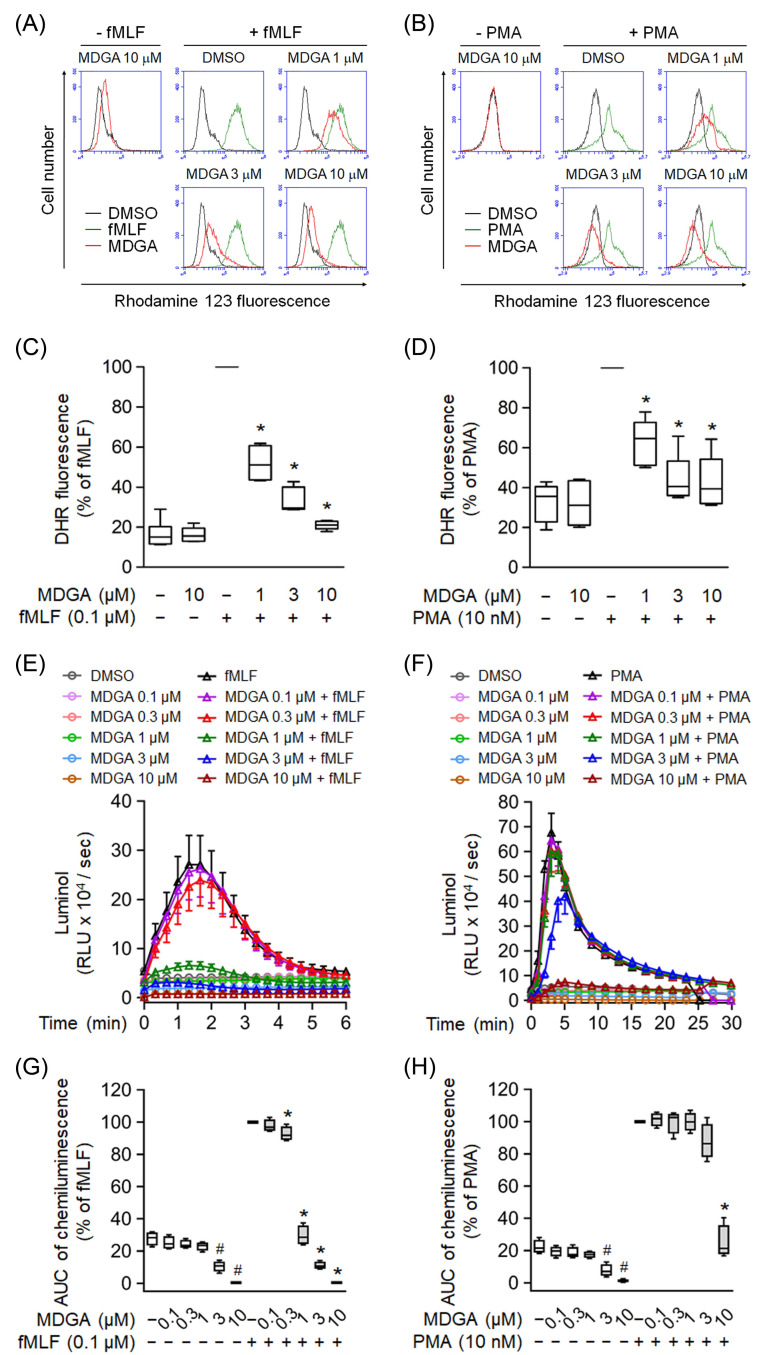
*meso*-Dihydroguaiaretic acid (MDGA) reduces reactive oxygen species (ROS) generation in activated human neutrophils. Dihydrorhodamine 123 (DHR123)- or luminol-incubated neutrophils were incubated with DMSO (0.1%, as control) or MDGA (0.1–10 μM), and activated by fMLF (0.1 μM) or PMA (10 nM). ROS production was detected by flow cytometry or chemiluminometer. The mean fluorescence intensity of (**A**,**B**) was quantified and shown in (**C**,**D**), respectively. The AUC value of (**E**,**F**) was quantified and shown in (**G**,**H**), respectively. The values are shown as the mean ± S.E.M. (*n* = 6). # *p* < 0.05 vs. non-stimulated control; * *p* < 0.05 vs. stimulated control.

Effects of MDGA on ROS production were further analyzed in human neutrophils. MDGA not only inhibited fMLF-induced intracellular ROS generation but also that induced by PMA in human neutrophils in concentration-dependent manners with IC_50_ values of 0.79 ± 0.26 μM and 3.57 ± 3.93 μM, respectively (Figure 2A–D). Similar results, MDGA inhibited total ROS generation in both fMLF- and PMA-induced human neutrophils (Figure 2E–H). Since MDGA did not inhibit PMA-induced superoxide anion generation (Figure 1D), we suppose that MDGA has a radical scavenging effect.

### 3.3. MDGA Exhibits a Free Radical Scavenging Effect

**Figure 3:**
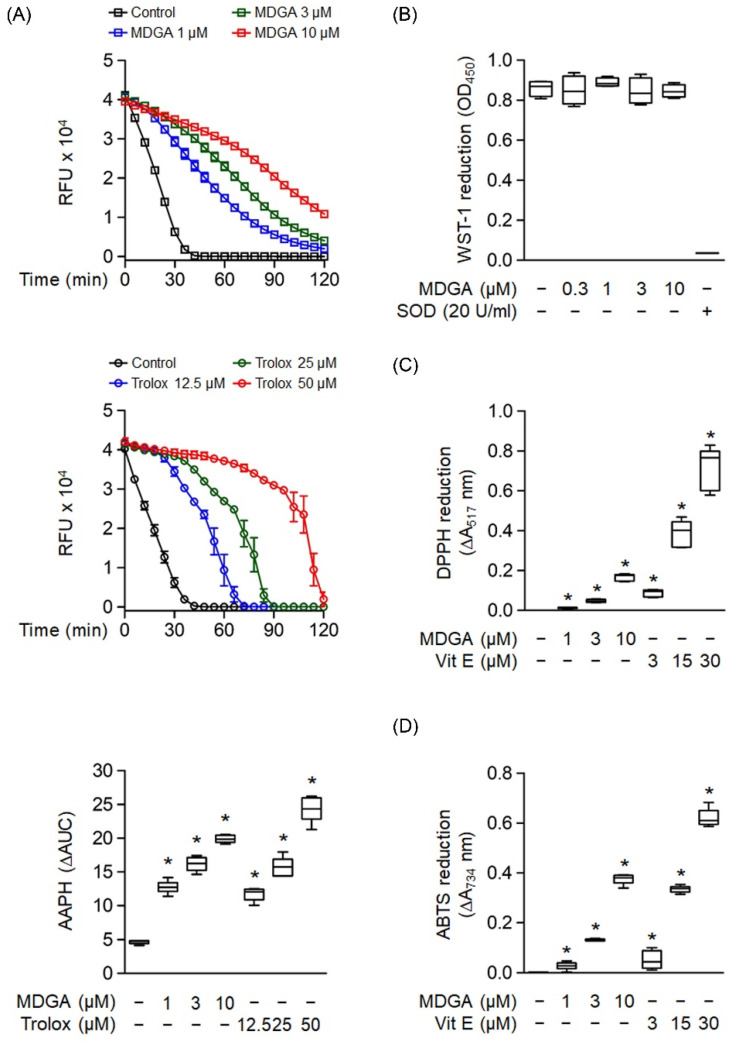
Radical scavenging effects of *meso*-dihydroguaiaretic acid (MDGA). (**A**) The change in absorbance of fluorescence curves of 2,2′-azobis(2-methylpropionamidine) dihydrochloride (AAPH) represented the decay of MDGA and Trolox (*n* = 6). The lower panel demonstrated quantitative fluorescence levels of MDGA and Trolox. (**B**) Reduction in WST-1 by xanthine/xanthine oxidase was measured spectrophotometrically at 450 nm. Superoxide dismutase (SOD) was used as a positive control (*n* = 4). (**C**) 1,1-Diphenyl-2-picrylhydrazyl radical (DPPH), was incubated with DMSO (0.1%, as control), MDGA (1, 3, and 10 μM), or vitamin E (Vit E; 3, 15, and 30 μM). Reduction in DPPH was calculated spectrophotometrically at 517 nm (n = 6). (**D**) 2,2′-Azino-bis(3-ethylbenzothiazoline-6-sulfonic acid) diammonium salt (ABTS) was incubated with DMSO (0.1%), MDGA (1, 3, and 10 μM), or Vit E (3, 15, and 30 μM). Reduction in ABTS was measured spectrophotometrically at 734 nm (*n* = 6). All data are expressed as mean ± S.E.M. * *p* < 0.05 vs. control.

Oxygen radical absorbance capacity assay was used to evaluate the ROS scavenging ability of MDGA (Figure 3A). MDGA exhibited ROS scavenging ability, as evidenced by comparing fluorescence decay against the background (Figure 3A, upper panel). Trolox, a water-soluble analog of vitamin E, was used as a positive control (Figure 3A, middle). The quantitative boxplot was shown in Figure 3A (lower). The cell-free xanthine/XO system was used to investigate whether MDGA had a direct O_2_^•−^ scavenging effect. Figure 3B shows that MDGA (0.3–10 μM) did not affect O_2_^•−^ produced by WST-1 reduction, where SOD was used as the positive control with a 95.9% reduction compared with the untreated control group. Moreover, MDGA exhibited RNS scavenging activity, as shown by DPPH and ABTS assays, which used vitamin E as the positive control (Figure 3C,D).

### 3.4. MDGA Reduces Elastase Release in Activated Human Neutrophils

**Figure 4:**
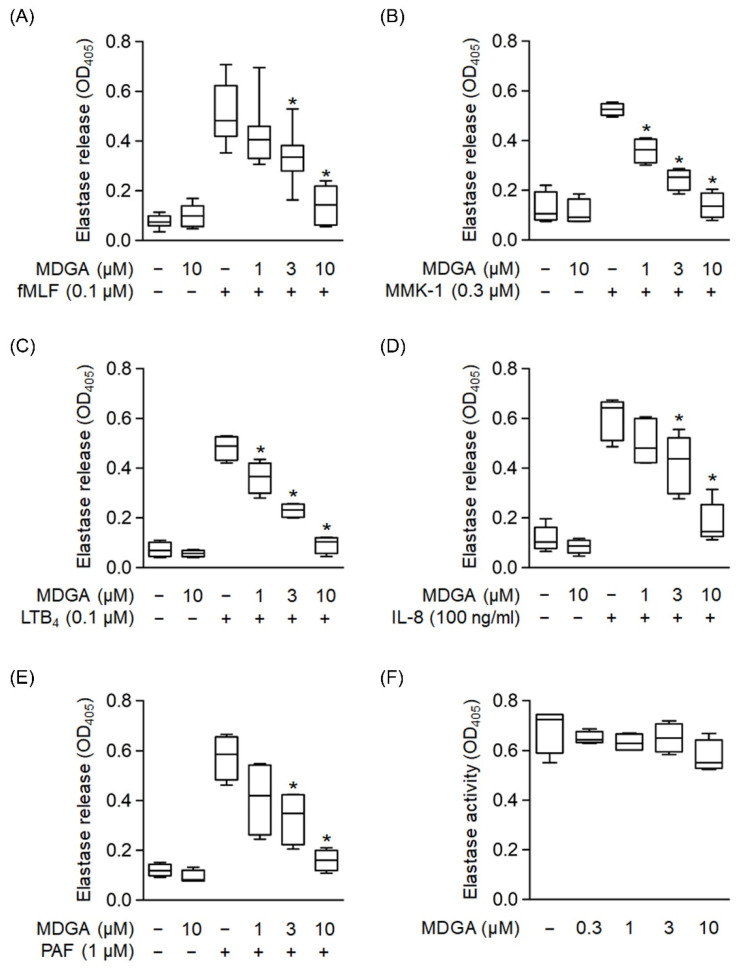
*meso*-Dihydroguaiaretic acid (MDGA) suppresses elastase release from stimulated human neutrophils. Human neutrophils were prepared with DMSO (0.1%) or MDGA (1, 3, and 10 μM) and then activated by (**A**) fMLF (0.1 μM) + CB (0.5 μg/mL), (**B**) MMK-1 (0.3 μM) + CB (0.5 μg/mL), (**C**) leukotriene B_4_ (LTB_4_, 0.1 μM) + CB (0.5 μg/mL), (**D**) interleukin-8 (IL-8, 100 ng/mL) + CB (0.5 μg/mL), or (**E**) platelet-activating factor (PAF, 1 μM) + CB (0.5 μg/mL). Elastase release was measured spectrophotometrically at 405 nm. All data are shown as the mean ± S.E.M. (*n* = 4–7). * *p* < 0.05 vs. stimulated control. (**F**) Human neutrophils were incubated with fMLF (0.1 μM) + CB (1.5 μg/mL) for 15 min. The elastase supernatant was obtained and then incubated with DMSO (0.1%) or MDGA (0.3–10 μM) for 2 min before the addition of substrate (100 μM). Elastase activity was measured spectrophotometrically at 405 nm. All data are shown as the mean ± S.E.M. (*n* = 4).

We used different reagents to activate neutrophils and determine whether MDGA inhibited elastase release. Elastase release was dose-dependently decreased by MDGA treatment upon fMLF, MMK-1, LTB_4_, IL-8, and PAF stimulation with IC_50_ values of 3.95 ± 1.31 μM, 1.32 ± 0.53 μM, 2.27 ± 0.6 μM, 3.91 ± 1.5 μM, and 2.22 ± 1.64 μM, respectively (Figure 4A–E). MDGA did not alter elastase release in resting human neutrophils. Additionally, MDGA did not directly inhibit elastase activity (Figure 4F). Based on these results, we suggest that the anti-inflammatory effects of MDGA in human neutrophils aim to inhibit the common downstream pathways of G protein-coupled receptors (GPCRs).

### 3.5. MDGA Suppresses CD11b Expression and Neutrophil Adhesion

**Figure 5:**
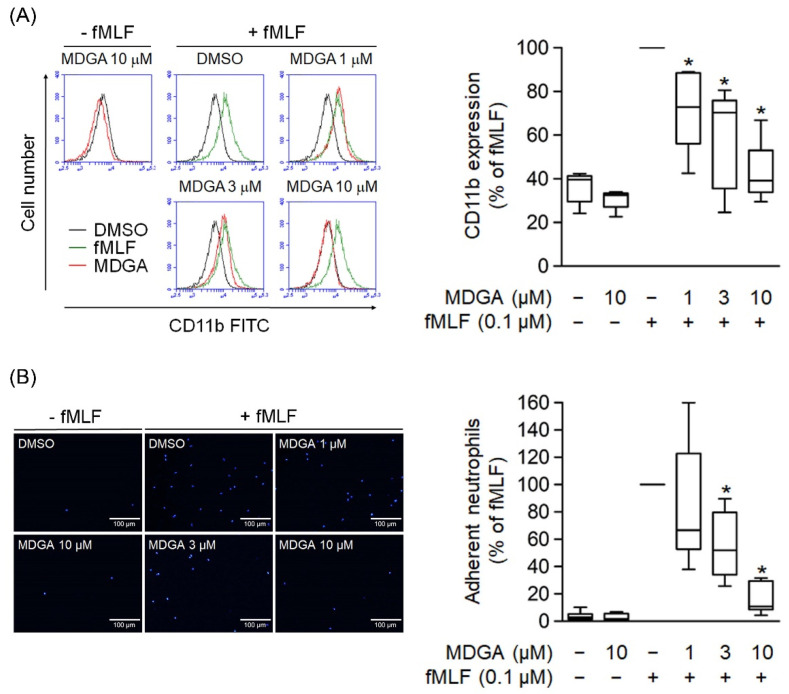
*meso*-Dihydroguaiaretic acid (MDGA) inhibits human neutrophil CD11b expression and adhesion. (**A**) After incubation with DMSO (0.1%, black line) or MDGA (1, 3, and 10 μM, red line), human neutrophils were stimulated by fMLF (green line). The mean fluorescence intensity was quantified and shown in the right panel. (*n* = 5). (**B**) Neutrophils were first stained with Hoechst 33342 and then incubated with DMSO (0.1%, as control) or MDGA (1, 3, and 10 μM) before fMLF activation. Stimulated neutrophils were co-cultured with bEnd.3 cells and the numbers of adherent neutrophils were calculated using microscopy. Bar, 100 μm. Adherent neutrophils were measured in the right panel. (*n* = 6). All data are shown as the mean ± S.E.M. * *p* < 0.05 vs. activated control.

The level of cell surface integrin plays a critical role in neutrophil adhesion during inflammation. Human neutrophils activated with fMLF resulted in the upregulation of cell surface CD11b integrin expression (Figure 5A). Similar to CD11b expression, cell adherent to endothelium was considerably increased in fMLF-activated neutrophils (Figure 5B). Furthermore, MDGA treatment significantly inhibited the fMLF-induced CD11b expression and cell adhesion (Figure 5).

### 3.6. MDGA Inhibits NET Formation

**Figure 6:**
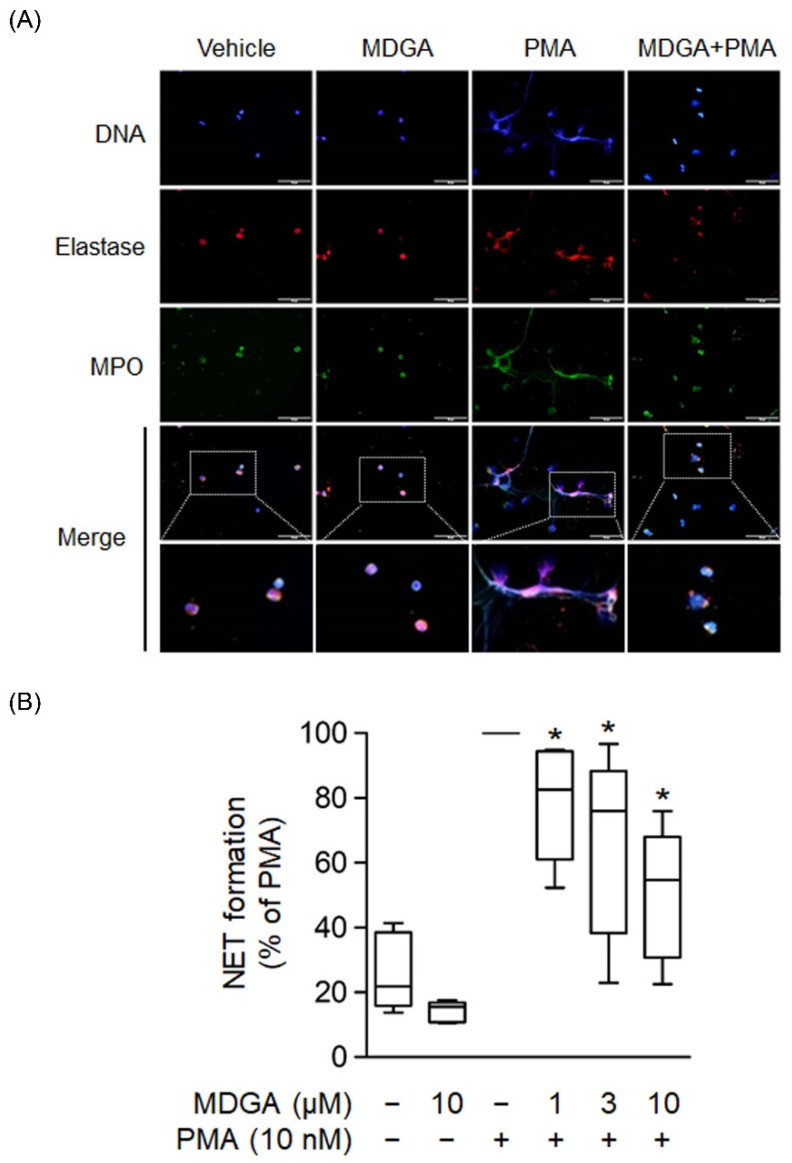
*meso*-Dihydroguaiaretic acid (MDGA) reduces NET formation. (**A**) Neutrophils incubated with DMSO (0.1%) or MDGA (10 μM) and stimulated with PMA (10 nM) were stained with anti-elastase (red) and anti-myeloperoxidase (MPO, green) antibodies. Hoechst 33342 (blue) demonstrated DNA. Scale bars are 50 μm (*n* = 3). Scar bar: 50 µm. (**B**) Neutrophils were incubated with DMSO (0.1%) or MDGA (1, 3, and 10 μM) and then stimulated with PMA. The amount of free DNA was measured by SYTOX Green. All data are shown as the mean ± S.E.M.* *p* < 0.05 vs. activated control (*n* = 5).

NETs are formed by de-condensed chromatin fibers with granular proteins such as elastase and MPO. Recent clinical and basic studies demonstrate that NETs play a significant pathogenic role in various inflammatory diseases and autoimmune disorders [39,40]. Human neutrophils were incubated with PMA, a protein C activator, for 2 h to induce NET formation, and the colocalization of extracellular DNA, MPO, and elastase was observed (Figure 6A). Figure 6A showed that MDGA inhibited PMA-induced formation of extracellular DNA, MPO, and elastase. Furthermore, NET formation quantification was assayed using a cell membrane-impermeable nucleic acid dye SYTOX Green [41]. The assays showed that MDGA inhibited NET formation in PMA-induced human neutrophils in a dose-dependent manner (Figure 6B).

### 3.7. MDGA Decreases Phosphorylation of ERK, JNK, and Akt Signaling

**Figure 7:**
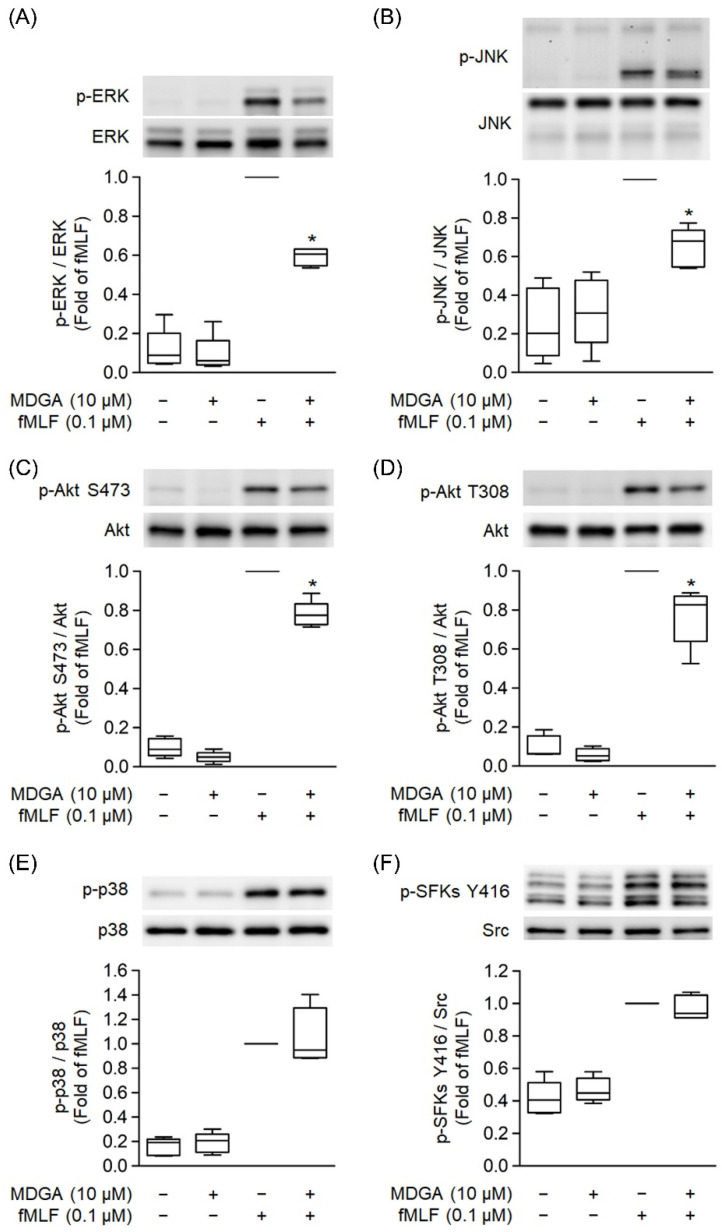
*meso*-Dihydroguaiaretic acid (MDGA) inhibits phosphorylation of ERK, JNK, and Akt, but not p38, SFKs in activated neutrophils. DMSO (0.1%) or MDGA (10 μM)-treated neutrophils were activated with or without fMLF. Phosphorylation of (**A**) ERK, (**B**) JNK, (**C**) Akt S473, (**D**) Akt T308, (**E**) p38, and (**F**) SFKs Y416 was assessed with immunoblotting using antibodies against the phosphorylated and native (total) forms of each protein. Blotted bands were analyzed with a densitometer, and the quantitative ratios of all samples were normalized to the corresponding total protein. All data are expressed as the mean ± S.E.M; (*n* = 5). * *p* < 0.05 vs. stimulated control.

MAPKs, Src family kinase (SFK), and Akt Phosphorylation play a significant role in GPCR agonist-induced human neutrophil activations [37,42]. fMLF significantly induced phosphorylation of ERK, JNK, p38, SFK, and Akt (Figure 7). MDGA treatment showed inhibitory effects on the phosphorylation of ERK as well as JNK and Akt in fMLF-induced human neutrophils (Figure 7A–D). However, fMLF-induced phosphorylation levels of p38 and SFKs were not altered by MDGA (Figure 7E,F).

### 3.8. MDGA Ameliorates LPS-Induced ARDS in BALB/c Mice

**Figure 8:**
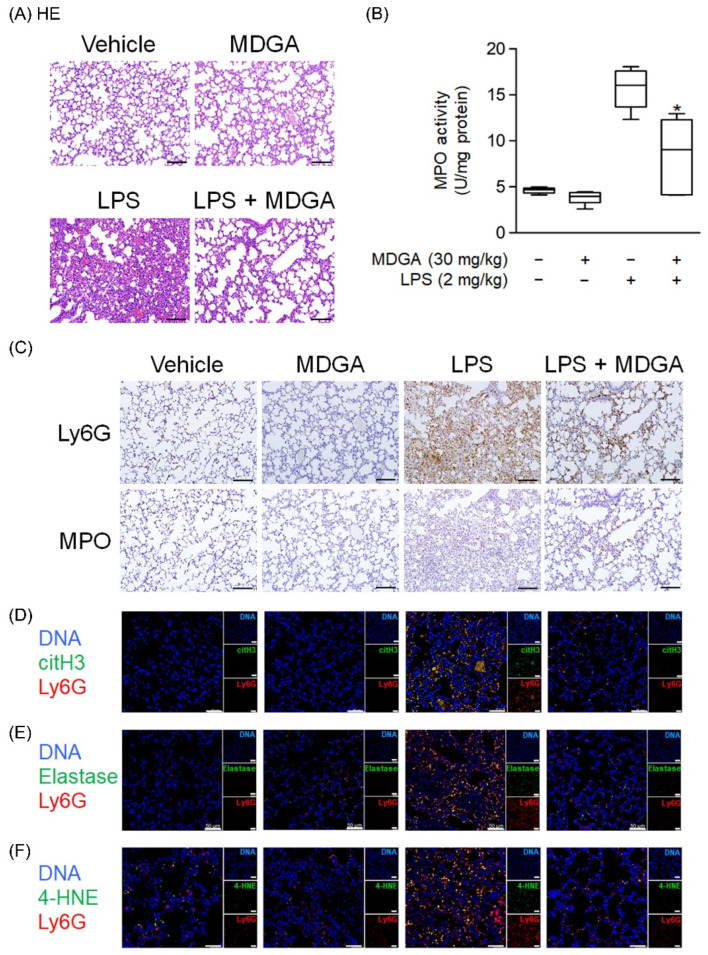
*meso*-Dihydroguaiaretic acid (MDGA) attenuates lipopolysaccharide (LPS)-induced neutrophilic lung inflammation. BALB/c mice were intraperitoneally treated with DMSO or MDGA (30 mg/kg) for 1 h, and LPS was administered intratracheally for 6 h. Mice were divided into four groups: vehicle, MDGA only, LPS only, and LPS with MDGA groups. (**A**) HE stains of the lung. (**B**) An assay to evaluate lung MPO activity. (**C**) Images of Ly6G and MPO in lung sections. Immunofluorescence staining of 4’,6-diamidino-2-phenylindole (DAPI), lymphocyte antigen 6 complex locus G6D (Ly6G), histone H3 (citH3) (**D**), elastase (**E**), and 4-HNE (**F**) in lung sections. Data are illustrated as the mean ± S.E.M. (*n* = 6). * *p* < 0.05 vs. the LPS group.

Endotracheal LPS administration (2 mg/kg) directly induced mouse ARDS. As shown in Figure 8A, pathological features such as hemorrhage and interstitial thickening were observed in the LPS-administered group by HE stains. MDGA treatment significantly improved these pathological changes in the lung. LPS induced increasing MPO level, which was reduced by MDGA (Figure 8B). Consistent with these results, Ly6G and MPO immunohistochemistry staining revealed that neutrophil infiltration was increased in LPS-administered groups, which was subsequently ameliorated by MDGA treatment (Figure 8C). Furthermore, MDGA treatment effectively reduced LPS-induced NET formation (Ly6G+citH3+ cell accumulation), elastase accumulation, and oxidative stress (4-HNE) (Figure 8D–F).

## 4. Discussion

**Figure 9:**
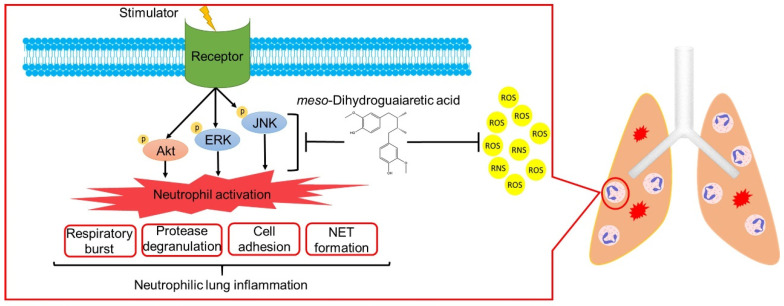
*meso*-Dihydroguaiaretic acid (MDGA) suppresses neutrophil respiratory burst, protease degranulation, cell adhesion, and NETs by inhibiting ERK, JNK, and Akt signaling and direct ROS scavenging effects. Furthermore, MDGA ameliorates ARDS in the LPS-induced mouse model.

ARDS is a high mortality systemic syndrome characterized by the acute onset of respiratory failure and hypoxemia. Neutrophils are most abundant in white blood cells and are the primary effectors in the innate immune system. Accumulating evidence indicates that ARDS is a key example of neutrophil-mediated tissue injury. Excessive neutrophil infiltration may cause lung injury due to increased superoxide anion generation, neutrophil elastase, MPO release, and NET formation [4,6,10]. Neutrophil counts reportedly significantly correlate with the disease severity of patients with ARDS associated with coronavirus disease 2019 (COVID-19) [43]. Clinically, no routine and effective pharmacological therapy exists for ARDS. Many scientists suggest that neutrophils can be a drug target for COVID-19 associated ARDS [6,44,45]. To the best of our knowledge, this is the first study to show that MDGA decreases neutrophilic lung inflammation by attenuating Akt/MAPK signaling (Figure 9). Taken together, MDGA could be a potential compound for ARDS treatment, which acts by inhibition of neutrophilic lung inflammation.

One of the functional characteristics of neutrophils is the activation of a powerful respiratory burst with ROS generation. In neutrophils, ROS exhibits antimicrobial activity and modulate immune response; however, in excess, ROS can lead to lung injury [7,46]. Hence, maintaining redox balance in the lung is important and could be a therapeutic strategy for ARDS. MDGA effectively inhibited the formation of superoxide anion in activated human neutrophils, but not in the cell-free xanthine/xanthine oxidase system, suggesting that MDGA inhibits neutrophil respiratory burst. MDGA did not inhibit PMA-induced superoxide anion generation in human neutrophils, therefore ruling out the effects of MDGA on the PKC-dependent downstream pathway. Phosphorylation of MAPKs and Akt plays a significant role in mediating GPCR-activators-induced neutrophil activations [33,47]. Our results showed that MDGA suppressed the phosphorylation of ERK, JNK, and Akt in fMLF-activated human neutrophils. Interestingly, MDGA inhibited PMA-induced ROS production in neutrophils, indicating that MDGA has a ROS scavenging effect. This effect may attribute to the high electron resonance property of phenoxyl substructure in MDGA [19,20]. In line with this result, MDGA showed direct ROS and RNS scavenging effects in cell-free systems. Taken together, MDGA can reduce oxidative stress by regulating neutrophil respiratory burst and direct scavenging ability.

Neutrophil elastase is one of the serine proteases protecting the host from pathogen invasion by fusing with phagolysosome inside neutrophils or releasing it to extracellular spaces [48]. However, neutrophil elastase also plays a role in tissue damage or remodeling [49] and is a key factor for the pathological development of pulmonary diseases. Pulmonary diseases, such as pneumonia [50], ARDS [51], and cystic fibrosis [52], are highly correlated with the expression of neutrophil elastase. Several animal studies indicate that the use of neutrophil elastase inhibitors can reverse the damage caused by neutrophil-mediated lung injury. Nevertheless, the role of neutrophil elastase inhibitors in ARDS treatment remains controversial [49]. Sivelestat, a neutrophil elastase inhibitor, together with a free radical scavenger, protects the lung tissue from neutrophilic damage in an LPS-induced ARDS model [53]. Our investigation indicates that MDGA can inhibit elastase release from neutrophils under different stimuli. Together with its oxidative stress-reducing effect, MDGA might be considered as a useful therapeutic agent for ARDS.

NETs, comprising de-condensed chromatin fibers with proteins such as histones, elastase, and MPO, contribute to various human diseases [54]. Excessive NET formation influences pulmonary microcirculation and induces disseminated lung injury, such as cystic fibrosis, asthma, chronic obstructive pulmonary disease, and ARDS [55]. Many studies indicate that NET formation is a therapeutic target for treating ARDS [6,44,45]. Within neutrophils, ROS and proteases can mediate NET formation. The inhibition of ROS formation, myeloperoxidase, and elastase activity is a useful strategy to attenuate NET formation [56,57]. MDGA effectively inhibits superoxide anion formation, ROS generation, and elastase release, as well as NET formation.

Neutrophil infiltration into the lung is a critical step in ARDS pathophysiology. When lung injury occurs, the circulating neutrophils migrate to the impaired lung via adhesion to the endothelium of lung vessels and then transmigrate to the alveolar space. CD11b/CD18, known as MAC1, is one of the integrins that facilitates neutrophil binding to endothelial cell surface molecules [58]. A sepsis-induced ARDS study has shown that MAC1 (CD11b/CD18) upregulation leads to neutrophil aggregation. Aggregated neutrophils create dead space in the pulmonary microcirculation, which could be ameliorated using a MAC1 inhibitor [59]. Our data indicate that MDGA ameliorates LPS-induced ARDS may be, in part, because of CD11b reduction with decreased neutrophil adhesion and recruitment.

Lignans are common second metabolites in vascular plants. Their structures are constructed with phenylpropane units and majorly classified into furofuran, furan, dibenzylbutane, and arylnaphthalene groups [18,20]. Our previous works showed the anti-neutrophilic activities of furofuran, furan, and arylnaphthalene lignans [60,61,62,63,64]. Here, we found MDGA, a dibenzylbutane-type lignan, not only exhibited greater anti-neutrophilic ability than previous lignans but also attenuated various neutrophilic functions induced by GPCR agonists. More importantly, MDGA inhibit NET formation. To our knowledge, no lignan was reported to inhibit NET formation before. These results indicate that MDGA contains a core bioactive fragment and serves as a lead for subsequent structural optimization.

ARDS is a critical illness caused by multiple pathological factors, such as sepsis, severe injury or burn, viral pneumonia, problems from inhaling substances like smoke or chemicals, and other serious illnesses. However, the severity of ARDS is associated with multisystem organ failure, leading to poor prognoses with high mortality rates. Neutrophil influx into the lungs plays a pathogenic role in ARDS through ROS production, elastase and myeloperoxidase release, and NET formation. A targeted approach to inhibit the function of neutrophils could theoretically mitigate neutrophil-dependent lung damage in ARDS patients. In conclusion, our findings that MDGA treatment attenuates the neutrophil inflammatory responses and an LPS-induced ARDS murine model suggest new therapeutic interventions for neutrophil-mediated diseases. Herein, MDGA might also be considered an adjunctive therapeutic agent to attenuate other viral inflammatory responses, such as influenza virus and coronavirus infection, causing severe ARDS complications.

## 5. Conclusions

MDGA, a natural lignan, significantly inhibited neutrophil respiratory burst, degranulation, adhesion, and NET formation as well as scavenged free radicals. MDGA effectively ameliorated neutrophil-associated ARDS in LPS-induced mice. Therefore, MDGA can act as a lead compound for developing new therapeutics to treat ARDS.

## Data Availability

Data is contained within the article.

## Abbreviations

AAPH: 2,2′-azobis(2-methylpropionamidine) dihydrochloride; ABTS, 2,2′-azino-bis(3-ethylbenzothiazoline-6-sulfonic acid) diammonium salt; ARDS, acute respiratory distress syndrome; BSA, bovine serum albumin; CB, cytochalasin B; DAPI, 4’,6-diamidino-2-phenylindole; DHR 123, dihydrorhodamine 123; DMSO, dimethyl sulfoxide; DPPH, 1,1-diphenyl-2-picrylhydrazyl radical; ERK, extracellular signal-regulated kinase; fMLF, *N*-Formyl-Met-Leu-Phe; HBSS, Hank’s balanced salts solution; HE, hematoxylin and eosin; 4-HNE, 4-hydroxynonenal; HRP, horseradish peroxidase; IL-8, Interleukin-8; JNK, c-Jun *N*-terminal kinase; LDH, lactate dehydrogenase; LPS, lipopolysaccharide; LTB_4_, leukotriene B_4_; Ly6G, lymphocyte antigen 6 complex locus G6D; MAPK, mitogen-activated protein kinase; MDGA, *meso*-dihydroguaiaretic acid; MMK-1, Leu-Glu-Ser-Ile-Phe-Arg-Ser-Leu-Leu-Phe-Arg-Val-Met; MPO, myeloperoxidase; NETs, neutrophil extracellular traps; PAF, platelet-activating factor; PBS, phosphate-buffered saline; PMA, phorbol 12-myristate 13-acetate; RNS, reactive nitrogen species; ROS, reactive oxygen species; SFK, Src family kinase; SOD, superoxide dismutase; WST-1, 2-(4-Iodophenyl)-3-(4-nitrophenyl)-5-(2,4-disulfophenyl)-2*H*-tetrazolium monosodium salt; XO, xanthine oxidase.
